# The spatial network of skeletal proteins in a stony coral

**DOI:** 10.1098/rsif.2020.0859

**Published:** 2021-02-24

**Authors:** Manjula P. Mummadisetti, Jeana L. Drake, Paul G. Falkowski

**Affiliations:** ^1^ Environmental Biophysics and Molecular Biology Program, Department of Marine and Coastal Sciences, Rutgers, The State University of New Jersey, 71 Dudley Rd, New Brunswick, NJ 08901, USA; ^2^ Department of Earth, Planetary, and Space Sciences, University of California, Los Angeles, 595 Charles E. Young Drive East, Los Angeles, CA 90095, USA; ^3^ Department of Marine Biology, University of Haifa, 199 Aba Khoushy Avenue, Mount Carmel, Haifa 2498838, Israel; ^4^ Department of Earth and Planetary Sciences, Rutgers, The State University of New Jersey, Piscataway, NJ 08854, USA

**Keywords:** biomineralization, coral acid-rich proteins, extracellular matrix proteins, protein cross-linking, bis(sulfosuccinimidyl)suberate, skeletal organic matrix

## Abstract

Coral skeletons are materials composed of inorganic aragonitic fibres and organic molecules including proteins, sugars and lipids that are highly organized to form a solid biomaterial upon which the animals live. The skeleton contains tens of proteins, all of which are encoded in the animal genome and secreted during the biomineralization process. While recent advances are revealing the functions and evolutionary history of some of these proteins, how they are spatially arranged in the skeleton is unknown. Using a combination of chemical cross-linking and high-resolution tandem mass spectrometry, we identify, for the first time, the spatial interactions of the proteins embedded within the skeleton of the stony coral *Stylophora pistillata*. Our subsequent network analysis revealed that several coral acid-rich proteins are invariably associated with carbonic anhydrase(s), alpha-collagen, cadherins and other calcium-binding proteins. These spatial arrangements clearly show that protein–protein interactions in coral skeletons are highly coordinated and are key to understanding the formation and persistence of coral skeletons through time.

## 1. Introduction

Stony corals (phylum Cnidaria, class Anthozoa) evolved over 485 Mya [1,2], and over geological time formed massive reefs in tropical and subtropical seas. Despite their geological and ecological importance, the biomineralization process in these organisms is poorly understood. Further, a complete understanding of how they will respond to climate change requires a deeper knowledge of the biomineralization process than currently exists [3]. Over the past decade, a core set of more than 100 proteins has been identified in coral skeletons [4–7]. This ‘skeletome’ [8] appears to be highly conserved across coral taxa. At the nanoscale level, these proteins and other biomolecules, termed the extracellular matrix (ECM) of the skeleton or more specifically the skeletal organic matrix (SOM), are hypothesized to control the biomineral deposition process, shape, size and three-dimensional organization [2,9,10], as well as the mechanical properties and plasticity of the biomineral material [11].

Proteomic analysis of the skeletome has identified acid-rich, metal-binding, framework and adhesion proteins [2,4–7]. Of these, acid-rich proteins and carbonic anhydrases are the most highly characterized [2,12,13]. Coral acid-rich proteins (CARPs; alternatively called skeletal aspartic acid-rich proteins (SAARPs) [5]) can precipitate calcium carbonate from unamended seawater [12] and modify the mineral polymorph, or differing crystal structures for minerals with the same chemical composition [14]; in the case of stony corals, this is predominantly aragonite [15]. In addition, the coral calcification process uses soluble bicarbonate ions as the primary source of carbonate [12,16]. In the biomineralization process, carbon dioxide is produced. Hence carbonic anhydrases—both membrane-bound *Stylophora pistillata* carbonic anhydrase (STPCA) and skeletal STPCA2 [13,17]—allow bicarbonate replenishment through the rapid conversion of carbon dioxide to bicarbonate [18,19]. Once formed, the aragonite crystals must be cemented to each other and to the underlying skeleton. Proteins responsible for the attachment processes include collagens, cadherins and other calcium-binding and adhesion proteins [4,20]; however, the spatial organization of these molecules within the skeleton is completely unknown.

Chemical cross-linking combined with mass spectrometry sequencing is a well-established approach to understanding the spatial organization of proteins. This approach does not suffer from some of the problems encountered when using immunological methods and can be used to study spatially close skeletal proteins [21,22]. Here, we probed the spatial interactions in the Indo-Pacific stony coral *S. pistillata,* using a classical, well-characterized cross-linker, bis(sulfosuccinimidyl)suberate (BS3), which tethers primary amines up to 11.4 Å apart in the intact skeleton [23]. Our results, across both experimental and biological replicates, are highly reproducible at the protein level and reveal that, while many different proteins are required to work together to create the optimal conditions for mineralization to occur, they are not located randomly but are spatially highly organized. Here, we begin to clarify the spatial patterning of the calcification space as a new mineral is formed between the living tissue of the animal and the substrate or older skeleton.

## 2. Results and Discussion

### 2.1. The interactome—a topological model

**Figure 1.**
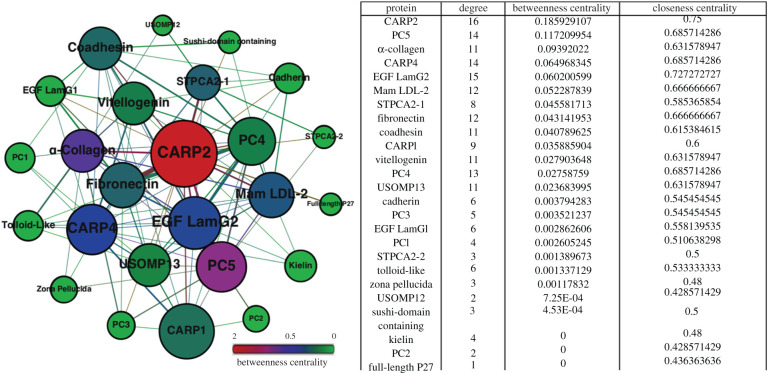
Interaction network of insoluble matrix proteins in *S. pistillata* skeleton. The network map shows individual proteins as nodes (circles) and their relations/interactions as edges (lines). These nodes and edges are scaled based on betweeness centrality, which measures the shortest paths among every pair of nodes in a network, which influences the colour of the node from green to red (green with the lowest interactions and red with the highest). The size of the node and the edge thickness depend on the number of connections between proteins.

The cross-link networks, termed ‘interactomes’ [24], reveal generalized patterns among skeleton building proteins (figure 1 and electronic supplementary material, SI appendix, figures S1–S4 and tables S1 and S2). These spatial interactions among the skeletal proteins are inferred upon treatment with BS3 to the cleaned, powdered coral skeleton. *S. pistillata* skeleton contains EDTA-soluble and -insoluble proteins in its aragonitic skeleton [4], of which individual coral skeletal proteins have the ability to precipitate calcium carbonate *in vitro* [14,25]. BS3 cross-linking impeded their ability to precipitate calcium carbonate (see below; electronic supplementary material, SI appendix, figure S10). For spatial interactions, the skeletal material is cross-linked and decalcified, and the cross-linked peptides, and hence proteins, are identified by high-resolution mass spectrometry. The separate interactomes for the soluble and insoluble proteins from the decalcified coral skeleton are presented in figure 1 and electronic supplementary material, SI appendix, figure S1, respectively.

The interaction networks are presented in the form of nodes, where each node represents a specific protein. The mathematically derived interactions based on the cross-linked proteins are inferred to represent true biochemical interactions responsible for skeletal formation. The networks are scaled based on betweenness centrality, which refers to the frequency at which each node (protein) occurs in the shortest paths between two other proteins (e.g. if a far-reaching node, protocadherin 2 (PC2), has direct connections only with CARP1 and PC5, it is now connected to many other proteins via CARP1 and PC5, and hence CARP1 and PC5 have greater betweenness centrality than PC2). Higher betweenness suggests the bridging property of a protein among clusters in a network. We also identify the centrality of proteins with another factor: closeness centrality, which refers to the frequency of interaction among two nodes as well as the interactions of the node to connect with the entire network. These topological measures are used to identify the key components in a network of multiple proteins.

The interactomes suggest that CARPs are spatially close to the two isoforms of the skeletal carbonic anhydrase STPCA2 and other CARPs, as well as adhesion and framework proteins including cadherins, vitellogenin, thrombospondin, epidermal growth factor (EGF) and laminin-G domain-containing (EGF LamG) proteins, and coadhesin. We also detected two uncharacterized proteins. The first, a von Willebrand factor type A (vWFA) domain-containing adhesion glycoprotein (SpiUSOMP 12 or USOMP12), was identified as a phosphopentothenoylcysteine decarboxylase [XP_022783323.1]; however, it does not contain any conserved domains found in other similar decarboxylases. It also has 28% sequence similarity to a protein in the stony coral *Acropora millepora,* annotated as collagen alpha-1(XIV) chain-like (XP_029187150.1) [6]. A second uncharacterized protein detected in our sequencing (SpiUSOMP 13 or USOMP13) appears to be a coral-specific protein [XP_022780049.1] [26]. In addition to the previously known skeletal carbonic anhydrase (STPCA2), we also identified an isoform and name it STPCA2-2. STPCA2-2 has an acidic insert region (electronic supplementary material, SI appendix, figure S5) comparable to STPCA2 (here, STPCA2-1).

### 2.2. The coral acid-rich proteins

Six CARPs/SAARPs have been characterized to date [4,6,12], and a seventh was sequenced in the present study. Most of these proteins contain more than 100 amino acids with approximately 30% or more of the sequence consisting of aspartic and/or glutamic acids, or share an orthologous relationship to such highly acidic proteins [27,28]. Owing to the ultra-high abundance of acidic residues, these proteins have isoelectric points between 3 and 4.5 and can precipitate aragonite in unamended seawater [12]. In this study, we found five of the seven previously known acid-rich proteins: CARP1, CARP2, CARP4/SAARP1, CARP5/SAARP2, an aspartic acid-rich protein identified here with high sequence similarity to partial-P27/acidic SOMP/SAARP3 [AGG36350.1] [4,5] and that we call full-length P27 (electronic supplementary material, SI appendix, figure S6), and a novel glutamic acid-rich protein that we have named CARP6 (electronic supplementary material, SI appendix, figure S7). This is also the first report of CARP1 and CARP2 being identified by mass spectrometry, although previous studies identified them by immunolocalization [29].

Our cross-linking analysis suggests that CARPs interact with each other in a variety of ways (electronic supplementary material, SI appendix, figures S3 and S4). CARP1 is the only CARP in close proximity to the E-rich CARP2 and D-rich CARP4. CARP1 has previously been shown, using immunolocalization and calcein staining of cell cultures of *S. pistillata*, to be a member of the ECM proteins and specifically adjacent to freshly formed CaCO_3_ particles [30]. Micro-Raman and nuclear magnetic resonance spectroscopy suggest that it and CARP3 bind to the aragonite phase in newly settled planulae [31]. CARP1 has seven EF-hand domains, which bind Ca^2+^, and has an 83% sequence similarity to calumenin [12], which is suggested for its role in bone morphogenetic protein (BMP)-2-dependent calcification and interactions with hydroxyapatite or calcite crystals [32].

Another highly acidic coral protein found to promote aragonite formation *in vitro* by binding to calcium is CARP2 [12]. Its glutamic acid residues have been detected in centres of calcification of *S. pistillata* spat [31]. It is the only known CARP which is upregulated in the pre-settled stage of planulae of the related pocilloporid coral *Pocillopora damicornis* [33]. This protein is suggested to precipitate a Mg-rich amorphous calcium carbonate (ACC), which is the first biomineral formed in the centres of calcification [15]. Our analysis found close spatial interactions of CARP2 with CARP1 and full-length P27, along with two carbonic anhydrases, STPCA2-1 and STPCA2-2 (electronic supplementary material, SI appendix, figure S4).

The glutamic acid-rich proteins CARP2 and the novel CARP6, both detected here for the first time by mass spectrometry, are the only CARPs to interact with an α-collagen, a framework protein that plays an important role in biomineralization. A similar glutamic acid-rich protein in vertebrate bones, bone sialoprotein (BSP), interacts with type 1 collagen [34] and promotes biomineralization in bone tissue by binding collagen and integrin [35]. BSP interacts with integrin at its arginine–glycine–aspartic acid sequence, and such an arginine–glycine–glutamic acid sequence is also seen in CARP2 (but not in other CARPs). BSP is a glycosylated phosphoprotein expressed exclusively in new mineralization zones [36], similar to CARP2 in aragonitic coral skeleton [31], and is responsible for hydroxyapatite nucleation and binding [37]. While the abundant negative charges from BSP's poly-E-rich regions are necessary for calcium-binding and initial interactions with collagen, the high-affinity binding with collagen is found in the protein's non-acidic regions [35,38], and BSP exhibits high-affinity binding to Ca^2+^ both *in vivo* and *in vitro* [34]. Based on the functional similarity, we performed sequence alignments of the N-terminus of BSP and CARP2 (electronic supplementary material, SI appendix, figure S8). Based on these alignments, the previously studied [38] BSP–collagen interaction sites are in close proximity to the cross-linked lysines of CARP2 and α-collagen (electronic supplementary material, SI appendix, figure S8). Another glutamic acid-rich protein which contains extended poly-E residues, as also seen in the novel CARP6 (electronic supplementary material, SI appendix, table S3), is known from vertebrate rod photoreceptors and has low-affinity binding to calcium but at a higher capacity [39]. Taken together, glutamic acid-rich regions probably play a major role to act as calcium buffers/concentrator at the centres of calcification.

The interaction of CARP2 with acidic carbonic anhydrase STPCA2-2 may have a specific role in centres of calcification, with the acidic domain of STPCA2-2 (electronic supplementary material, SI appendix, figure S5) contributing to the initial mineralization and binding to calcium ions upon its interaction with CARP2, while CARP2 (although acid rich) assists in protein interactions and increased ACC nanoparticle accumulation.

The interactions of CARP4 with CARP5 and CARP5 with CARP6 are especially interesting. These three CARPs have stretches of glutamic acid or aspartic acids (electronic supplementary material, SI appendix, figure S7), suggesting a similar role of CARP4/5 and CARP6 in calcium binding/concentrating during the biomineralization process. Based on their sequences (electronic supplementary material, SI appendix, figure S7), these three CARPs are intrinsically disordered [40,41]. The presence of such highly disordered proteins in mineralization is seen widely across the tree of life and yet their role is not well understood [4,6,34].

### 2.3. Carbonic anhydrases—STPCA2 and STPCA2-2

In addition to CARPs, another well-studied protein found in the coral skeleton is carbonic anhydrase. Previous immunolocalization studies have identified the widespread distribution of the carbonic anhydrase STPCA2 in the skeleton [4,29]. The *S. pistillata* genome encodes 16 carbonic anhydrases, of which three (STPCA, STPCA2 and STPCA3) are characterized for their chemical properties [42]. Proteomic studies of the *S. pistillata* skeleton, however, have only found STPCA2 to date [4,7] and a different carbonic anhydrase was detected in *Acropora digitifera* skeleton [6] but not in *A. millepora* [5]. We found a second isoform of STPCA2, here named STPCA2-2. These STPCA2 isoforms have a high sequence identity of about 74% except at their N-termini and at the insert region of STPCA2-2 (electronic supplementary material, SI appendix, figure S5). This region contains an abundance of acidic residues and appears to be specific to the two STPCA2 isoforms identified so far compared with the rest of the α-carbonic anhydrase superfamily as it is absent in mammalian carbonic anhydrases and the other cellular coral carbonic anhydrases (electronic supplementary material, SI appendix, figure S5).

Since STPCA2 isoforms contain evolutionarily conserved domains compared with the rest of the skeletal proteins, we modelled these two isoforms (STPCA2-1 and STPCA2-2) using I-TASSER [43]. All lysines involved in inter-protein interactions, based on our cross-linking work, from both STPCA2 isoforms appear at the surface of the proteins' proposed tertiary structures (electronic supplementary material, SI appendix, figure S9), suggesting that the STPCA2 structure may evolutionarily support a highly positively charged surface (electronic supplementary material, SI appendix, figure S9); this would allow its interactions with negatively charged domains (e.g. CARPs).

Protein cross-linking results in the reduction/loss of protein flexibility and therefore function [44]. This is consistent with our results from *in vitro* calcium carbonate precipitation assays using a calcite-promoting seawater medium to which we added cross-linked SOM, un-cross-linked SOM or no protein (electronic supplementary material, SI appendix, figure S10). The pair of reacting residues within a protein (lysines in this case for BS3) must be within the reacting distance and at the same time accessible to the cross-linker for the proteins to become cross-linked. This non-cleavable linkage makes the local region of the protein static with subsequent loss of flexibility, dynamic properties and protein function [45,46]. We observed that, when EDTA-solubilized SOM proteins were incubated in seawater, precipitates containing calcium and magnesium were formed (electronic supplementary material, SI appendix, figure S10); however, when cross-linked- and then EDTA-solubilized SOM proteins were incubated in seawater, such precipitates were not formed. This confirms that cross-linking restrains SOM proteins and the dynamic behaviour required for their function of aragonite precipitation is subsequently lost.

**Figure 2.**
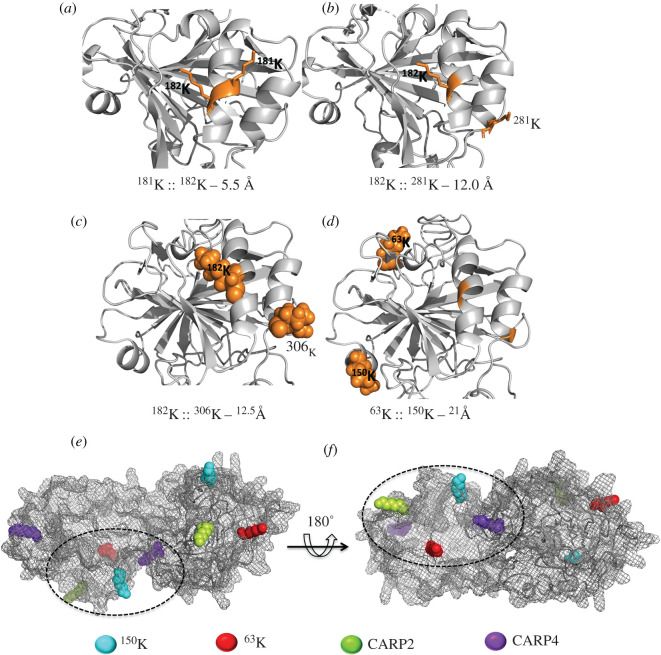
Interactions within STPCA2 isoforms. Carbonic anhydrase (STPCA2-1 and STPCA2-2) protein models were generated by I-TASSER. (*a*,*b*) The intra-molecular cross-links identified for STPCA2-1 by mass spectrometry are mapped onto these models, shown in orange sticks. (*c*,*d*) STPCA2-2; sphere representation of cross-linked residues in orange. Distances (below *a*–*d*) between cross-linked residues were measured in PyMOL. (*e*,*f*) Molecular docking of ^63^K and ^150^K from two STPCA2 isoforms. The lysines from STPCA2 interacting with CARP2 and CARP4, when mapped onto the STPCA2 docking model, show that all four residues are spatially close.

We then mapped the STPCA2 cross-link pairs onto their modelled structures and found that all the intra-STPCA2 cross-link pairs are located near each other and are on the same side of the folded protein (figure 2). Three of the four cross-linked pairs in the models are approximately 11.4 Å apart, strongly suggesting that BS3 reacts with native proteins in the skeletal environment and does not produce random cross-linked products. The fourth cross-link pair (^63^K::^150^K) has residues approximately 20 Å apart in the models (figure 2*d*), which could be for two reasons: these residues lie in the highly flexible coiled regions of STPCA2-2 (which is evident from the I-TASSER structure; figure 2) and/or these residues are derived from the STPCA2 isoforms. We tested this second hypothesis through molecular docking of the two identified STPCA2 isoforms (figure 2*e*,*f*). Molecular docking is a method to model two or more proteins (protein–ligand) at their interaction sites, either using predictive algorithms or from experimental data. In our study, we used experimentally identified cross-linked residues ^63^K:: ^150^K (since Lys-63 is present in both isoforms) as the interaction site of the two STPCA2 isoforms modelled in the interactive docking prediction program ZDOCK, which applies unison of shape complementarity and electrostatics to statistically score and generate models with high structural stability and predictive accuracy. As shown in figure 2*e*,*f*, once the STPCA2 isoforms are docked at STPCA2-1 ^63^K::STPCA2-2 ^150^K, these residues are within the cross-linking distance of BS3. Additionally, this interaction also brings the interacting residues of STPCA2 within the cross-linking distance of CARP2 and CARP4, which would otherwise be far apart (electronic supplementary material, SI appendix, figure S11).

Although we could not capture a direct interaction between CARP2 and CARP4, it would seem necessary for the transition from ACC to aragonitic fine needles in the coral skeleton. While most CARP genes are upregulated after the settlement of coral planulae, CARP2 is the only CARP gene that is highly expressed in the pre-settled stage and is downregulated post-settlement in *Pocillopora damicornis* [31,47]. CARP2 has also recently been found associated with the ACC phase and was suggested to be involved in the formation of initial mineral precursors [31]; this is consistent with our cross-linking interaction between CARP2 and the framework protein α-collagen.

### 2.4. Structural and calcium-binding domain-containing proteins

Several transmembrane domain-containing proteins that become part of the skeleton include cadherin-like and other calcium-binding proteins. One such class of proteins is von Willebrand factor (vWF) proteins. The vWFA domain is seen in a large family of adhesion glycoproteins, whose structures are stabilized upon calcium binding [48]. These proteins enhance mineralization of bones *in vitro* [49], suggesting their role in the initial mineralization process during skeleton development. Genes for these proteins have previously been studied in pre- and post-settled larvae from *P. damicornis* [33] and *A. millepora* [50] and are upregulated in the pre-settled stage of coral larvae before mineralization commences. The vWFA proteins identified in our work include thrombospondin, USOMP12 and collagen. Thrombospondin contains a highly conserved protein-binding domain (TSP-1) and has previously been suggested for its role in biomineral remodelling in higher organisms [51]. The α-collagen probably plays an important role in controlling mineralization. Previous *in vitro* studies suggest its role in amorphous-phase mineral formation, which requires infiltration of the mineral into fibrils followed by their organization into oriented crystals [52]. α-Collagens are highly abundant, fibrous, insoluble and ECM proteins in eukaryotes and are known for their structural role. Several studies show *in vitro* nucleation and mineral formation in the presence of collagen, with the interaction of positive and negative charges on the proteins being suggested as a mechanism for mineral growth in solution [53]. We observed spatial interactions from vWFA domain-containing proteins such as thrombospondin, USOMP12 and collagen with CARPs and the STPCA2 isoforms.

The von Willebrand factor type D (vWFD) domain-containing proteins are another set of proteins that bind calcium and are an important structural component of the skeletal ECM. The skeletal proteins seen in our study with this domain include sushi domain-containing protein [AGG36340] and vitellogenin [XP_022779720.1]. Vitellogenin is notable for its interaction with lipopolysaccharides, peptidoglycans and polysaccharides such as glucan and laminarin by pattern recognition, and belongs to a group of multivalent pattern recognition receptor binding proteins that have been suggested for their role in innate immune defence [54]. Vitellogenin is also a precursor of egg yolk protein, helping to provide nutrients to developing embryos by its properties of recognizing proteins and lipids. Such pleiotropic roles of vitellogenin in fighting bacterial infections and its protein and lipid interactions may be important for coral skeleton formation. We found interactions of sushi domain-containing protein with coadhesin (which has vWFA- and FA58C-carbohydrate-binding domains), MAM-and-LDL domain-containing protein and CARP2 (figure 1, electronic supplementary material, SI appendix, figures S1 and S2), whereas vitellogenin interacts with several adhesion proteins (coadhesin, fibronectin, collagen, EGF LamG, protocadherin) and CARP4. Based on our observations of interactions of vWF domain-containing proteins with other adhesion proteins, and their expression in early stages of calcifying spat [33], it is clear that they play an important role in framework building, cell adhesion, and protein–protein, protein–lipid and protein–crystal interactions during the skeleton development process in corals.

We observed several other calcium-binding proteins, including the following. (i) Kielin-like and EGF LamG domain-containing proteins which contain one or more LamG domains that, in addition to binding calcium, perform a variety of functions, including adhesion and migration. (ii) MAM-and-LDL domain-containing proteins, which, as extracellular proteins, impart properties of homo-oligomerization and adhesion. (iii) The five sequences PC1–PC5 were also detected by mass spectrometry (electronic supplementary material, SI appendix, table S3). We compared these sequences with the skeletal protocadherin-like protein from *A. millepora* [JT JT011093.1] (electronic supplementary material, SI appendix, figure S13), which is predicted as a 450 kDa protein. Based on our alignment analysis, four out of five of the *S. pistillata* protocadherin sequences (PC1–PC4) have a very high sequence similarity to *A. millepora* protocadherin and they are each other's reciprocal best blast hit between the two species. We therefore propose that the sequences PC1–PC4 are actually part of a larger cadherin orthologous to the *A. millepora* skeletal protocadherin-like protein that was incompletely predicted in *S. pistillata*. We further note that, with their large number of extracellular cadherin repeats, EGF-like and LamG domains and cytoplasmic catenin-binding domain, these orthologous cadherins observed in *S. pistillata* [4], *A. millepora* [6] and *A. digitifera* [5] skeleton are likely to be classical cadherins [55]. (iv) A CUB (complement C1r/C1s, Uegf, Bmp1) domain-containing tolloid-like protein was also observed in our analysis. Tolloid-like proteins are known for their role in orchestrating the formation of ECM, patterning the biomineral and BMP signalling in morphogenetic processes [56]. Binding to these structural proteins as described above would allow the highly acidic proteins to be arrayed in an ordered fashion in the calcifying space for tighter control by corals over biomineralization.

To the extent that we can generalize patterns of protein interactions across biomineralizing taxa outside of Cnidaria, our results suggest that highly acidic mineralization-related proteins such as aspein, dentins and the SIBLING family of proteins [57] would also be associated with other acidic proteins [4,6,58] and carbonic anhydrases. The cementing proteins will depend on the biomineral that is precipitated. However, based on the limited number of proteomic analyses available at this time, we suggest that collagens and cadherin-like proteins would be broadly distributed in metazoan biominerals.

### 2.5. Concluding working model

**Figure 3.**
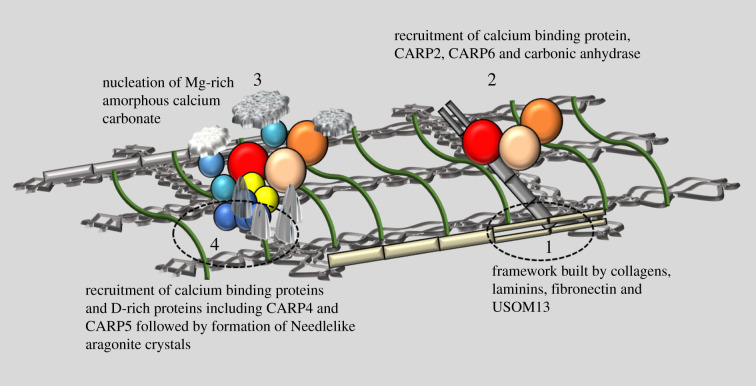
Working model of biomineralization. Based on previous research and our interactome analyses presented here, we suggest the following working model of stony coral biomineralization. 1. Skeletal vWF domain-containing proteins collagen, USOMP13 and laminins form a structural framework. 2. Glutamic acid-rich proteins CARP2 and CARP6 bind to collagen in the presence of STPCA2-2 (carbonic anhydrase). 3. Initiation of ACC phase formation, binding of other adhesion and calcium-binding proteins along with aspartic acid-rich proteins CARP4 and CARP5. 4. Formation of needle-like aragonite crystals. Confirmation of the timing of each step remains to be resolved.

A working model for the spatial organization of SOM proteins in the stony coral skeleton is shown in figure 3. We propose that the major ECM-forming skeletal proteins that contribute to the formation of the organic framework of the calcifying space (vWFA, vWFD family of proteins) first structure the microenvironment of the calcifying space. This is followed by their interaction with glutamic acid-rich protein CARP2 (and potentially CARP6) and carbonic anhydrases. Our study identified interactions of ECM-forming framework proteins with CARP2 and carbonic anhydrases. Previous expression studies indicate that vWFA, vWFD domain-containing proteins along with CARP2 are highly expressed during the initial, newly released stage of larval development [33]. The known characteristics of these proteins suggest that they lead to the formation of an initial ACC phase, followed by the recruitment of other adhesion and Ca^2+^-binding proteins (such as cadherins), a matrix-patterning tolloid-like protein, a BMP signalling protein and aspartic acid-rich proteins (e.g. CARP4 and CARP5). This is consistent with the late-stage expression of CARP4 and CARP5 in the larval development as settled calcifying spat [33]. Our interactome analysis supports that the calcium-binding proteins interact directly with acid-rich proteins or indirectly via cadherins and other adhesion proteins. The presence of such skeletal ECM-forming proteins (framework and adhesion proteins) along with matrix-patterning proteins would be necessary to provide polarity, organizing the CARPs and other acid-rich (i.e. disordered) proteins in the calcifying space during the biomineralization process [59].

## 3. Material and Methods

Fully detailed methods can be found in electronic supplementary material, Methods.

### 3.1. Study organism

Fragments of *S. pistillata* were grown in an in-house 800 litre flow-through system.

### 3.2. Cleaning coral skeleton

Skeletons were soaked overnight in 3% (wt/vol) sodium hypochlorite, copiously rinsed in deionized water and dried overnight at 37°C. Dried skeletons were ground to less than 150 µm diameter and then bleached, rinsed and dried again. The dried powder was further ground to less than 60 µm.

### 3.3 Skeletal organic matrix extraction

Cleaned skeletal powder for replicate samples (5 g each) was decalcified in 0.5 M EDTA at room temperature while shaking. Insoluble material was pelleted and washed in water followed by two washes in 10 mM phosphate-buffered saline (PBS), pH 8.0, 10 µM CaCl_2_; the final pellets were resuspended in 100 mM PBS, pH 8.0, 10 µM CaCl_2_. The EDTA-soluble fractions were concentrated and washed twice in 10 mM PBS, pH 8.0, 10 µM CaCl_2_ and then once in 100 mM PBS, pH 8.0, 10 µM CaCl_2_ in 10 kDa Amicon centrifugal filter units (Millipore Sigma). At this stage, samples were concentrated to 200 µl and stored at −20**°**C. Data from three biological replicates and experimental replicates were pooled together to generate our final results.

### 3.4. Protein cross-linking

Cross-linking reactions using BS3 (Proteochem) dissolved in mass spectrometry-grade water were performed on the cleaned skeletal powders at 5 mM and 10 mM BS3 in PBS pH 7.4 for 1 h at room temperature. The reaction was stopped using 40 mM ammonium bicarbonate for 20 min at room temperature. Pelleted skeleton containing cross-linked proteins was rinsed to remove any unreacted cross-linker before dissolution in EDTA as described above.

### 3.5. Electrophoresis and protein digestion

Cross-linked proteins were stacked into gels by sodium dodecyl sulfate polyacrylamide gel electrophoresis; the entire band was excised from the gel, fixed in 50% ethanol and 5% acetic acid for 30 min, washed in water several times, washed in a solution of 25 mM ammonium bicarbonate and 50% acetonitrile several times [60], dried in a SpeedVac and stored frozen until enzymatic digestion. Immediately prior to mass spectrometric analysis, proteins were treated with dithiothreitol and iodo-acetic acid followed by trypsin digestion [60].

### 3.6. Mass spectrometry

Tryptic digests were resolved on a C18 reversed-phase column. Spectrometry was performed on a QE Exactive Orbitrap instrument. Proteins were identified by both the MassMatrix search engine and X!Tandem using a FASTA search library created from the *S. pistillata* transcriptome [61]. Identified proteins with at least two unique peptides and a *p-*value ≤10E^−10^ were retained to create a custom cross-link search database. Cross-linked peptides with a *p-*value **≤**10E^−3^ were considered for further analysis.

### 3.7. Cross-link peptide search

Mass spectrometry generated files were first analysed by MassMatrix version 2.4.2 using a ≤1% false discovery rate on a decoy search. Then, peptide matches from the first step were searched using MassMatrix's search algorithm to identify the cross-linked peptides based on the BS3 range constraint (11.4 Å). In the third step, the quality of each peptide match was measured by a single *p-*value calculated from a probabilistic score obtained from the number of matched peaks, a probabilistic score obtained from the ion intensity distribution of matched peaks and a probabilistic score obtained from consecutiveness of matched peaks.

### 3.8. Protein modelling and network maps

Carbonic anhydrase models were generated using I-TASSER by homology modelling [43]. The PDB files of protein structure generated by I-TASSER were visualized in PyMOL. These PDB files were also used for docking analysis performed using ZDOCK. The results from BS3 cross-linking are presented as network maps. These are created with the network analysis and visualization program Gephi [62].

### 3.9. *In vitro* calcium carbonate precipitation assay

This assay was performed to identify if the ECM proteins were functional after application of protein cross-linker and their extraction from the coral skeleton (electronic supplementary material, SI appendix, figure S11). Briefly, 10 µg of total proteins from the EDTA-solubilized skeletal powder (with and without BS3 treatment) was incubated in artificial seawater in sterile six-well plates. Precipitates were collected from the surface of the wells, diluted and imaged under scanning electron microscopy. A detailed method for this assay is included in the supplementary methods.

## Data accessibility

All data are available in the article, electronic supplementary material and the Pride repository. The mass spectrometry proteomics data have been deposited to the ProteomeXchange Consortium via the PRIDE [63] partner repository with the dataset identifier PXD020076.
